# Morphometric Analysis of Placenta and Fetal Doppler Indices in Normal and High-Risk Pregnancies

**DOI:** 10.7759/cureus.61663

**Published:** 2024-06-04

**Authors:** G Mahalinga, KV Rajasekhar, M Venkateshwar Reddy, S. Saravana Kumar, Syed Khaja Waheeduddin

**Affiliations:** 1 Department of Anatomy, Meenakshi Academy of Higher Education and Research, Chennai, IND; 2 Department of Radiology, Meenakshi Medical College Hospital and Research Institute, Chennai, IND; 3 Department of Anatomy, Sri Venkata Sai (SVS) Medical College and Hospital, Mahabubnagar, IND; 4 Department of Anatomy, Meenakshi Medical College Hospital and Research Institute, Chennai, IND

**Keywords:** preeclampsia toxemia, placenta, intrauterine growth restriction, doppler indices, hypertension, gestational diabetes, high-risk pregnancies

## Abstract

Background

High-risk pregnancies, encompassing pregnancy-induced hypertension (PIH), gestational diabetes mellitus (GDM), preeclampsia toxemia (PET), and intrauterine growth restriction (IUGR), represent intricate medical challenges with potential repercussions for maternal and fetal health. This research undertakes a comprehensive comparative investigation into the variations of Doppler indices and placental parameters within the context of these high-risk conditions when juxtaposed against pregnancies characterized as normal.

Methodology

Employing a rigorous cross-sectional study design, a diverse cohort of pregnant individuals with gestational diabetes, IUGR, PIH, and preeclampsia was meticulously assembled. Additionally, a group of normal pregnant women served as the comparative reference. Doppler ultrasound assessments, viz, pulsatility index (PI), were carefully performed to estimate blood flow velocities within critical maternal and fetal vessels, while placental parameters were meticulously quantified, encompassing dimensions, vascular architecture, and morphological features.

Results

Except in the GDM group, all high-risk groups had reduced estimated placental weight and actual birth weight than normal pregnant women. All high-risk groups showed a highly significant elevation of the PI of the umbilical artery and PI of the middle cerebral artery (MCA) than normal but the PI of MCA was significantly reduced in the PET group than in normal individuals. The cerebro-placental ratio in the GDM and IUGR groups revealed markedly greater values, whereas PET showed lower values. IUGR and PIH groups showed a substantial reduction in the fetal birth weight. All high-risk groups (GDM, IUGR, PIH, and PET) showed a highly significant reduction in luminal area umbilical artery 1 than the normal pregnant women. In IUGR, marginal placental insertion was very high, followed by GDM and PET groups.

Conclusions

This study reveals that Doppler indices, placental parameters, newborn weight, and their related ratios may be utilized to anticipate gestation difficulties and gain insight into the pathophysiology of problematic conceptions.

## Introduction

Pregnancy is a critical bliss in a woman’s life, which almost every time can bring forth various neuropsychological dissension during the gestational period. This study aimed to determine the prevalence and outcomes of high-risk pregnancy and the factors associated with it among antenatal women in urban, semi-urban, and rural health care centers in the Telangana state of south India. Almost 15% of all pregnant women can develop potentially life-threatening complications. As a result, identification of high-risk pregnancies at the earliest stage is useful in directing appropriate intervention. Approximately 22% of women suffer from high-risk pregnancy during their pregnancy [1]. Preeclampsia, gestational diabetes mellitus (GDM), and small for gestational age (SGA) are frequent issues associated with pregnancy. Globally, the recurrent problematic conceptions have progressively increased by 5% to 10% for SGA [2], 2% to 5% for preeclampsia [3], and 2% to 13% for GDM [4].

Recent research has demonstrated that developmental abnormalities in the fetus and placental disorders can complicate pregnancies. Of note, the placenta, a transitory structure for regulating nourishment from the mother to the offspring, affects birth weight [5]. Recent research has indicated a vivid correlation between its weight and fetal birth weight [6,7]. According to several studies, factors related to the placenta have a significant influence on fetal developmental restrictions, and all macroscopic and microscopic pathological abnormalities suggest that vascular injury is the root cause of the restricted flow of blood [2].

When compared to individuals born with normal development, intrauterine growth restriction (IUGR) is an indication of a perinatal risk that leads to morbidity and death. The occurrence of IUGR differs significantly between various groups of people. Its frequency is close to 33% in newborns who weigh less than 2,500 g at delivery. Economic growth is also correlated with the occurrence of IUGR, which is substantially lower in wealthy nations (4%-8%) than in poor ones (6%-30%) [8]. Certain established indicators of the possibility of IUGR include infections, preeclampsia, hypertension, diabetes mellitus, cardiovascular disorders, low socioeconomic position, and placental insufficiency [9]. According to reports, of all the arteries examined by Doppler ultrasonography, the middle cerebral artery (MCA) and the umbilical artery (UA) are the most accessible as well as repeatable. The MCA of the fetuses has been carefully inspected to determine the fetal blood flow rate [10].

The pulsatility index (PI) of the MCA and UA ratio, often called the cerebro-placental (CP) ratio, is a valuable indicator of the health of the fetus. A lower CP ratio, as opposed to MCA or UA Doppler indices alone, shows relative redistribution of the blood flow to cerebral irrigation and is thought to increase precision in forecasting challenges and adverse outcomes [11]. This proportion is now being used more frequently in monitoring the conceptus of danger by repeating the Doppler examination frequently. Even though these Doppler indices have reference ranges in the literature from the West, there are few studies of a comparable nature conducted among the Indian population [12,13]. The peripheral resistance of the blood arteries is measured by the PI. An increase in resistance in the distal segments of the vessels may be indicated by higher PI, which denotes hypoperfusion in the area [14].

Independent indicators of unfavorable perinatal outcomes include low birth weight and the weight of the placenta. The etiology of both increased as well as decreased placental weight remains unsatisfactory. This study evaluates the correlation between placental and umbilical cord parameters as very little information was observed concerning Doppler indices in association with placental morphometry and luminal diameter of umbilical vessels.

This study undertakes a thorough investigation into the interplay between these high-risk conditions and their impact on Doppler indices and placental parameters by examining a diverse cohort of pregnant individuals from different socioeconomic spectrums afflicted by these complications. This study aims to uncover potential associations that could advance the understanding of their underlying mechanisms and clinical implications.

## Materials and methods

Study design

This observational, cross-sectional study was conducted from October 20, 2021, to May 12, 2023, in the Department of Anatomy, with the collaboration of the Departments of Radiology, Obstetrics and Gynaecology, and Paediatrics at tertiary care and teaching hospitals. At different phases of pregnancy, participants underwent a series of ultrasound/Doppler scans for research purposes. Both patients and researchers were unaware of the scan results. The study scan findings were made accessible after delivery. Placental and neonatal parameters were obtained after parturition.

Study population

The study included pregnant women from different clinical groups, i.e., GDM, IUGR, pregnancy-induced hypertension (PIH), preeclampsia toxemia (PET), and a control group of normal pregnancies. Women who participated in all planned prenatal investigation scans and gave birth to a live child following 36 weeks of conception met the inclusion criteria for this study among those with high-risk pregnancies. Women who left the study early or whose fetuses were found to have abnormalities were excluded.

Sample size

The sample size was assessed depending on power analysis to ensure sufficient statistical significance. It accounted for the number of clinical groups, anticipated dropout rates, and the desired level of statistical power.

Doppler assessment

All individuals who met the inclusion criteria were recruited in this investigation. Parameters were assessed utilizing the LOGIQ P5 duplex Doppler ultrasound equipment, which has a curvilinear low-frequency transducer. The technical features included spectral frequency, frequency, filter medium, sample volume, and PRF-4-5MH [15].

MCA PI, UA PI, and CP ratio

Participants were assessed by employing a 3.5 MHz curvilinear transducer for duplex Doppler once the biometry results were confirmed. Doppler waveforms from the UA and the fetal MCA were recorded across three successive cardiac cycles. The fetus was asleep and apneic when the patients were evaluated while they were lying semi-recumbent. Spectral waveforms were created with the use of a medium filter and a 4 mm sample volume [15].

MCA PI

The MCA is the closest to the probe and was found using the color Doppler each time. A 4 mm sample volume was used to get a spectral trace from the MCA immediately after it was formed. Every time, it was ensured that the angle of insonation was between 0 and 60 degrees. Both human and automated PI evaluations were performed throughout three successive cardiac cycles. The parameters were repeated, and two interpretations that had similar findings were recorded for this study [15].

UA PI

The UA was located in every instance employing color Doppler. A spectral trace was created utilizing a 4 mm sample volume from the umbilical cord’s free loop. If it was not able to locate the free loop of the UC, the placental implantation of the chord was tracked. From 0 to 60 degrees, the angle of insonation was maintained constant. The PI was computed both automatically and manually across three successive cardiac cycles. The measurements were repeated, and the final two interpretations that gave similar findings were recorded [15].

MCA PI/UA PI Ratio

CP ratio, a computation comparing the MCA PI to UA PI, was performed for each patient after confirming the technical accuracy of the examination and measurements.

Follow-up studies

Neonatal and Placental Morphometry

**Figure 1:**
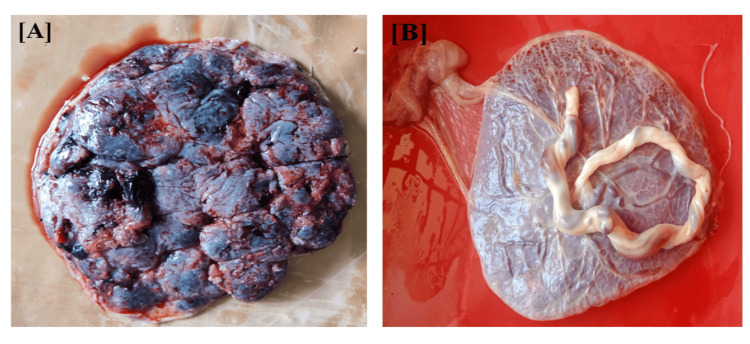
Placenta. (A) Maternal surface showing cotyledons. (B) Fetal surface along with amnion.

Neonatal measurements included birth weight collected immediately after birth. Placental measurements included estimated weight, actual weight, diameter, thickness, number of cotyledons, fetoplacental ratio, placental coefficient, placental shape, and cord insertion. The length of the umbilical cord was measured, and umbilical cord samples were collected for histopathological examination of umbilical vessels (Figure 1).

Histopathology

The tissue of the umbilical cord was fixed in a 10% formalin solution and then sectioned at 4 μ thickness. Employing hematoxylin and eosin, tissue sections were stained and observed for histopathological alterations and measured luminal area [16]. Staining was done with normal hematoxylin and eosin, slides were observed using biological microscope Leica DM500, and luminal areas were measured using Leica LAS software.

Statistical analysis

The statistical evaluations were performed utilizing SPSS version 21 (IBM Corp., Armonk, NY, USA/). The Student’s t-test was performed for statistical comparisons between two categories. A P-value <0.05 was considered significant. One-way analysis of variance (ANOVA) was employed to evaluate variations among the groups. The mean and standard deviation were used to describe quantitative data of high-risk pregnancies with GDM, PIH, PET, and IUGR with controls having a normal distribution.

## Results

Estimated placental weight by ultrasonography

**Figure 2:**
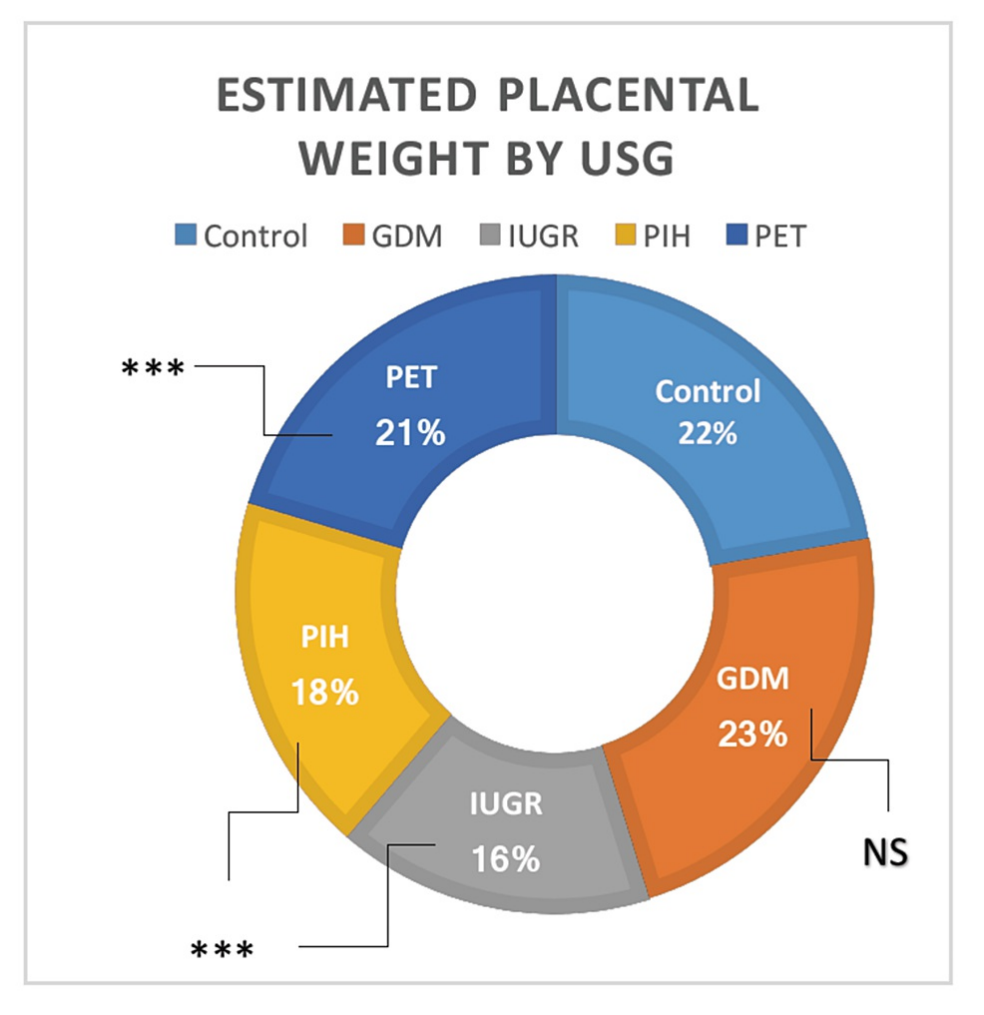
Parameters of estimated placental weight by USG in high-risk groups in comparison with the control group. USG = ultrasonography; GDM = gestational diabetes mellitus; PIH = pregnancy-induced hypertension; PET = preeclampsia toxemia; IUGR = intrauterine growth restriction

This study’s findings exhibited considerable variation among the groups according to one-way ANOVA. Except for the GDM group, all groups (IUGR, PIH, and PET) showed a highly significant reduction in estimated placental weight than the normal pregnant women (t = 8.87, p = 0.00001; t = 7.94, p = 0.00001; and t = 2.45, p = 0.007464, respectively) (Table 1). Using Tukey’s honestly significant difference post hoc analysis, the major variation was further evaluated and determined to be considerable (p ≤ 0.05). The estimated placental weight was examined between the groups. It was significantly less in IUGR, PIH, and PET groups than in GDM (t = 7.134, p = 0.00001; t = 6.38, p = 0.00001; and t = 2.18, p = 0.015888, respectively). The IUGR group showed the least estimated placental weight than the rest of the groups, followed by the PIH and PET groups. The GDM vs. IUGR, GDM vs. PIH, GDM vs. PET, IUGR vs. PIH, IUGR vs. PET, and PIH vs. PET showed statistically significant difference (t = 7.13, p = 0.00001; t = 6.38; p = 0.00001; t = 2.18, p = 0.015888; t = 3.30, p = 0.000699; t = 3.24, p = 0.001013; and t = 2.04, p = 0.022133, respectively) (Figure 2).

**Table 1:** Statistical comparisons of high-risk pregnancies across various groups with controls. The statistical significance is shown by the scripted stars (*): ***: p = 0.00001, **: p = 0.01, *: p = 0.05, NS = non-significant. USG = ultrasonography; ANOVA = analysis of variance

Statistical comparisons with the ANOVA test	Gestational diabetes mellitus		Intrauterine growth restriction		Pregnancy-induced hypertension		Preeclampsia toxemia	
	P-value	Significant differences	P-value	Significant differences	P-value	Significant differences	P-value	Significant differences
Estimated placental weight by USG by volume	0.09939	Non-significant	0.00001	Highly significant	0.00001	Highly significant	0.007464	Highly significant
Actual placental weight in grams	0.094051	Non-significant	0.00001	Highly significant	0.00001	Highly significant	0.00587	Significant
Fetoplacental ratio	0.447334	Non-significant	0.127079	Non-significant	0.308426	Non-significant	0.417154	Non-significant
Placental coefficient	0.274445	Non-significant	0.000498	Highly significant	0.026725	Significant	0.053983	Non-significant
Umbilical artery pulsatility index	0.00001	Highly significant	0.00001	Highly significant	0.00001	Highly significant	0.00001	Highly significant
Middle cerebral artery pulsatility index	0.00001	Highly significant	0.00001	Highly significant	0.00001	Highly significant	0.00001	Highly significant
Cerebro-placental ratio	0.000448	Highly significant	0.000609	Highly significant	0.39399	Non-significant	0.00001	Highly significant

Actual placental weight in grams

**Figure 3:**
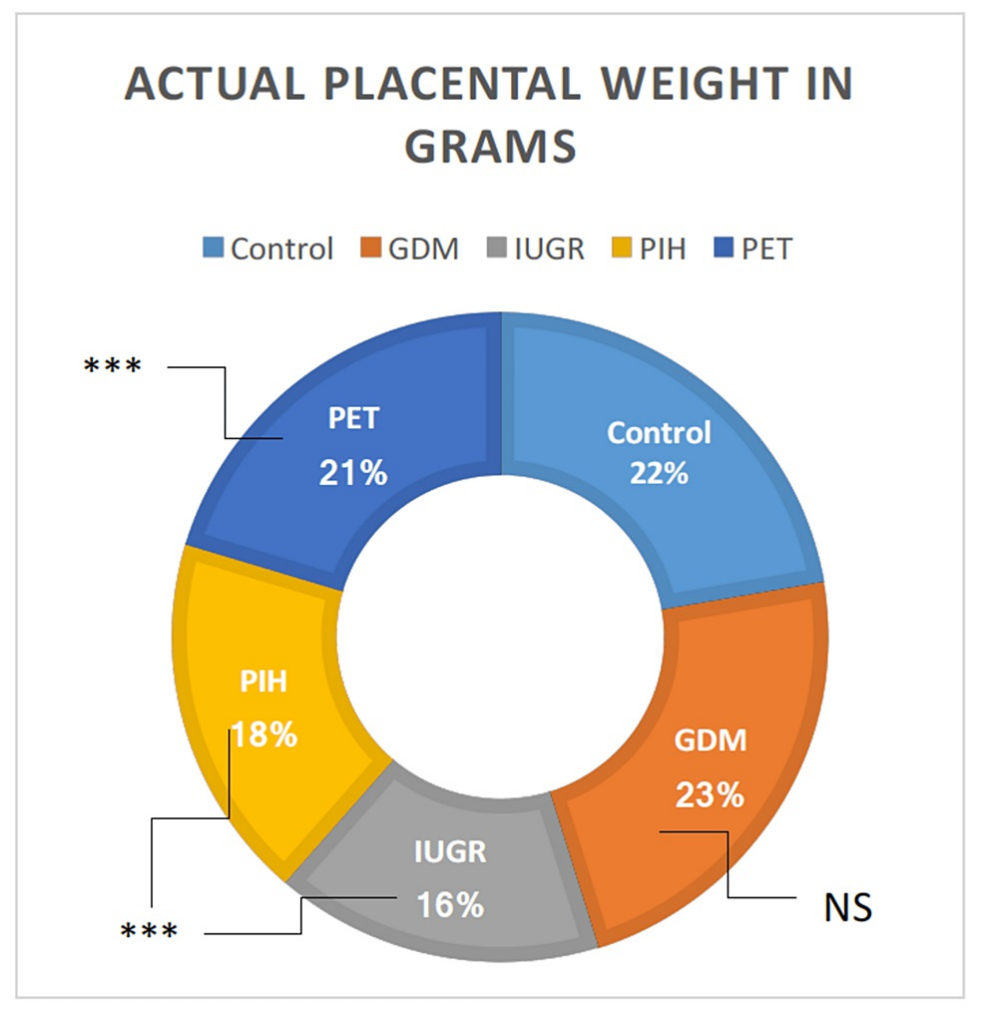
Parameters of actual placental weight in grams in high-risk groups in comparison with the control group. GDM = gestational diabetes mellitus; PIH = pregnancy-induced hypertension; PET = preeclampsia toxemia; IUGR = intrauterine growth restriction

The findings of the one-way ANOVA showed a noteworthy difference among the groups in the actual weight of the placenta. Except for the GDM group, all groups (IUGR, PIH, and PET) showed a highly significant reduction in the actual placental weight than the normal pregnant women (t = 8.81, p = 0.00001; t = 8.02, p = 0.00001; and t = 2.53, p = 0.00587, respectively) (Table 2). The actual placental weight was examined between the groups. It was significantly less in IUGR, PIH, and PET groups than in GDM (t = 7.134, p = 0.00001; t = 6.38, p = 0.00001; and t = 2.18, p = 0.015888, respectively). The IUGR group showed the least actual placental weight than the rest of the groups, followed by the PIH and PET groups. The GDM vs. IUGR, GDM vs. PIH, GDM vs. PET, IUGR vs. PIH, IUGR vs. PET, and PIH vs. PET showed statistically significant differences (t = 7.18, p = 0.00001; t = 6.50, p = 0.00001; t = 2.28, p = 0.012412; t = 3.08, p = 0.001389; t = 3.23, p = 0.001029; and t = 2.06, p = 0.021054. respectively) (Figure 3).

**Table 2:** Comparative investigation showing mean ± STDEV of Doppler indices and placental parameters within the context of high-risk pregnancies with controls. STDEV = mean and standard deviation; USG = ultrasonography

Groups	Control		Gestational diabetes mellitus		Intrauterine growth restriction		Pregnancy-induced hypertension		Preeclampsia toxemia	
	Mean	STDEV	Mean	STDEV	Mean	STDEV	Mean	STDEV	Mean	STDEV
Estimated placental weight by USG by volume	577.645	89.877	595.578	122.201	418	51.844	475.516	81.750	528.966	167.430
Actual placental weight in grams	514.295	79.581	530.468	106.439	373.423	50.085	422.566	74.176	470	144.586
Fetoplacental ratio	6.174	4.043	6.105	1.339	5.262	1.040	6.439	1.368	6.017	1.733
Placental coefficient	0.173	0.032	0.170	0.035	0.196	0.034	0.163	0.040	0.186	0.076
Umbilical artery pulsatility index	1.164	0.068	1.2753	0.0959	1.2980	0.1267	1.285	0.086	1.275	0.1071
Middle cerebral artery pulsatility index	1.2096	0.0680	1.3573	0.0880	1.3884	0.0807	1.3316	0.1036	1.1113	0.1186
Cerebro-placental ratio	1.0403	0.0467	1.0676	0.0843	1.0746	0.0760	1.0381	0.0779	0.872	0.0723
Fetal birth weight in grams	3,081.85	2,010.61	3136.69	411.11	1,941.15	315.04	2644.5	361.64	2,598.96	328.34

Fetoplacental ratio

**Figure 4:**
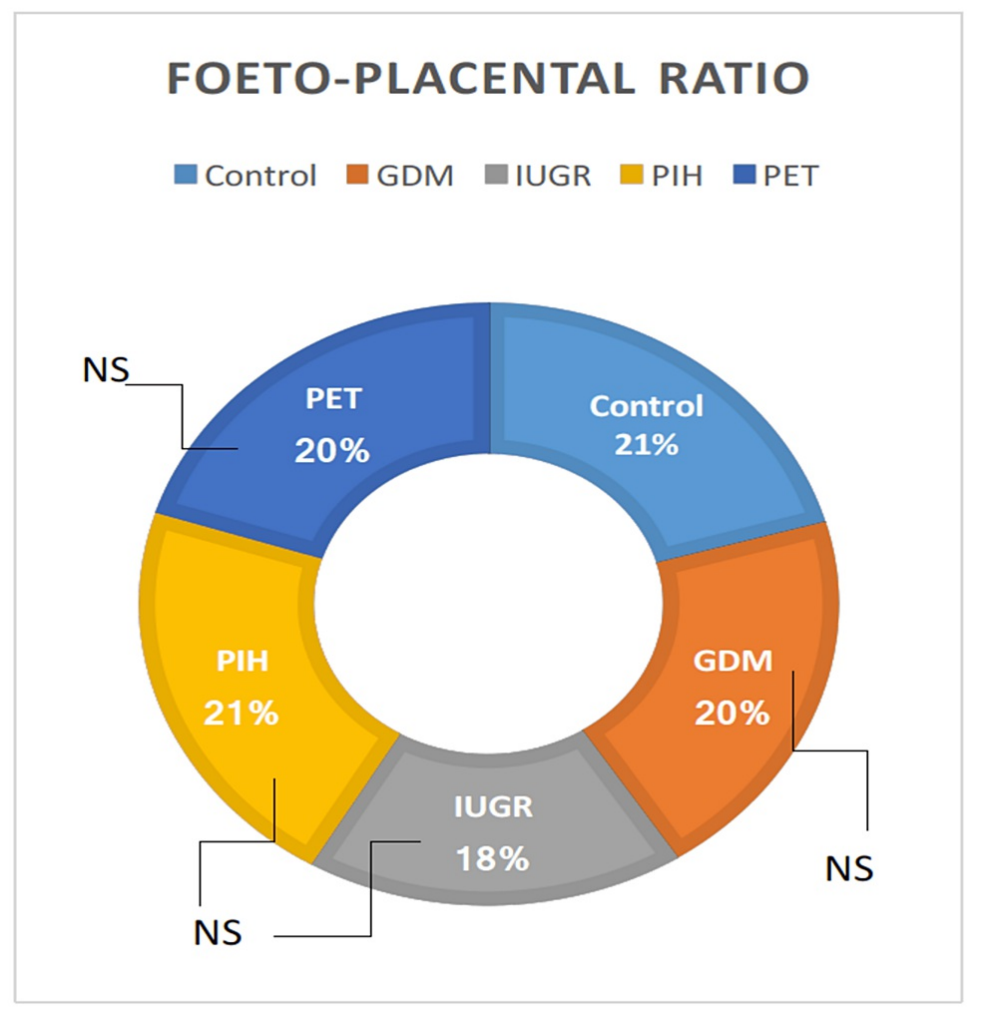
Parameters of the fetoplacental ratio in high-risk groups in comparison with the control group. GDM = gestational diabetes mellitus; PIH = pregnancy-induced hypertension; PET = preeclampsia toxemia; IUGR = intrauterine growth restriction

The difference in the fetoplacental ratio among the groups was not significant, as determined by one-way ANOVA (Table 1). No high-risk group showed a significant difference when compared with normal individuals. However, GDM vs. IUGR, IUGR vs. PIH, and IUGR vs. PET exhibited statistical differences (t = 2.87, p = 0.447334; t = 3.91, p = 0.127079; t = 1.93, p = 0.308426, respectively), whereas GDM vs. PIH, GDM vs PET and PIH vs. PET showed significant difference (Figure 4).

Placental coefficient

**Figure 5:**
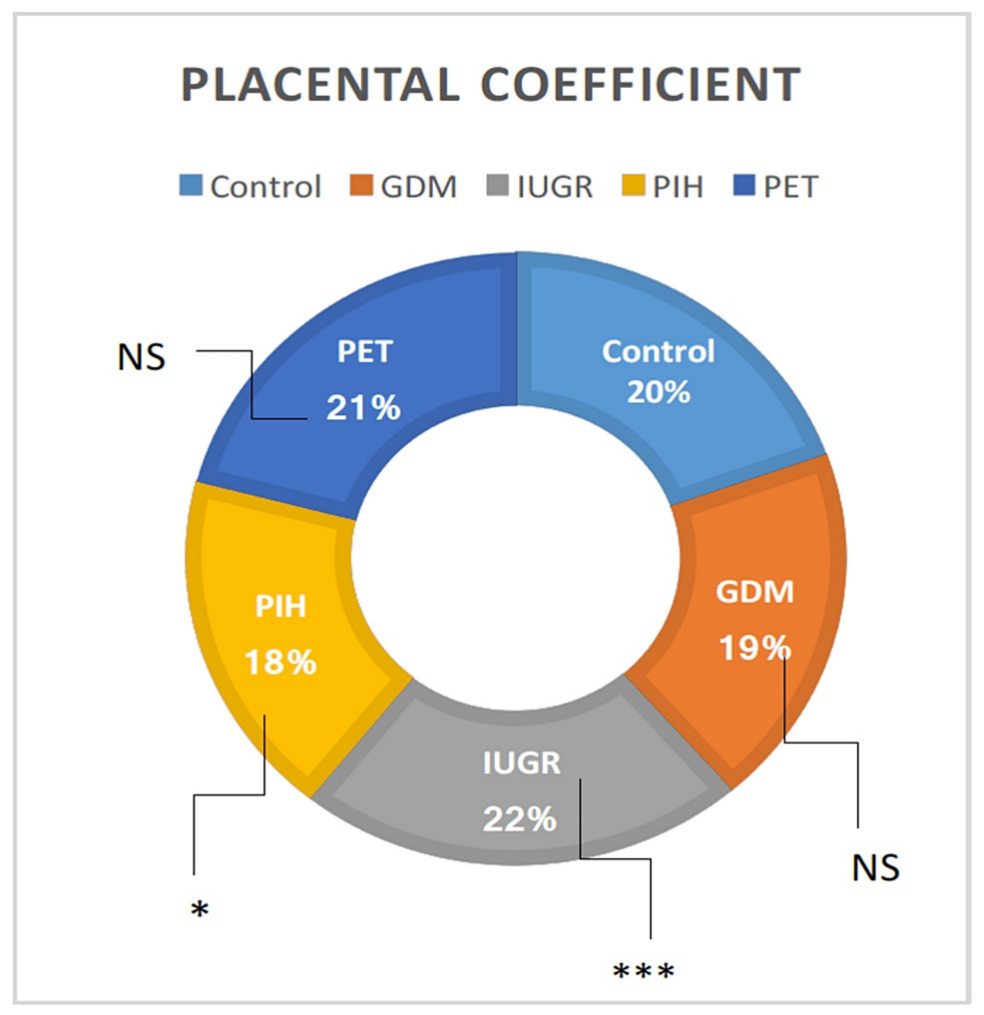
Parameters of placental coefficient in high-risk groups in comparison with the control group. GDM = gestational diabetes mellitus; PIH = pregnancy-induced hypertension; PET = preeclampsia toxemia; IUGR = intrauterine growth restriction

A statistically considerable difference in the placental coefficient was observed among the groups using one-way ANOVA. When compared between the groups, t-test results exhibited no difference in the GDM and PET cohort than the normal individuals. However, the PIH and PET groups showed significantly higher placental coefficients when compared with normal individuals (t = 3.33, p = 0.000498 and t = 1.93, p = 0.026725, respectively) (Table 1). The least placental coefficient was found in PIH group individuals. The GDM vs. IUGR, IUGR vs. PIH, and PIH vs. PET showed considerable differences (t = 3.15, p = 0.053983; t = 3.56, p = 0.000307; and t = 1.82922, p = 0.035377, respectively) whereas GDM vs. PIH, GDM vs. PET, and IUGR vs. PET did not exhibit considerable differences (Figure 5).

UA PI

**Figure 6:**
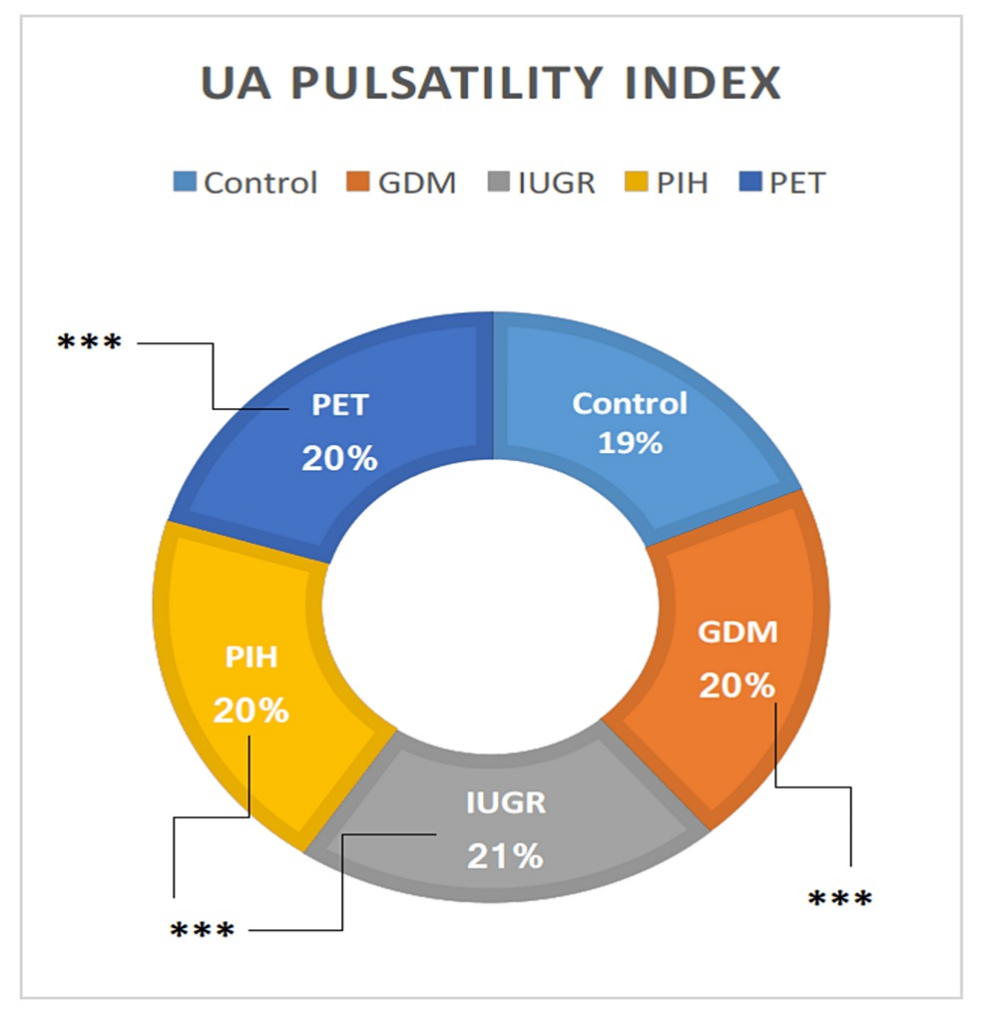
Doppler indices of UA in high-risk groups in comparison with the control group. UA = umbilical artery; GDM = gestational diabetes mellitus; PIH = pregnancy-induced hypertension; PET = preeclampsia toxemia; IUGR = intrauterine growth restriction

The ANOVA findings showed that there was a considerable variation in the UA PI among the groups. All high-risk groups (GDM, IUGR, PIH, and PET) showed highly significant (t = 10.32, p = 0.00001; t = 8.41, p = 0.00001; t = 11.39, p = 0.00001; and t = 7.66, p = 0.00001, respectively) elevation in UA PI than the control group (Table 1). UA PI was examined between the cohorts. There was no considerable distinction between the high-risk clusters (Figure 6).

MCA PI

**Figure 7:**
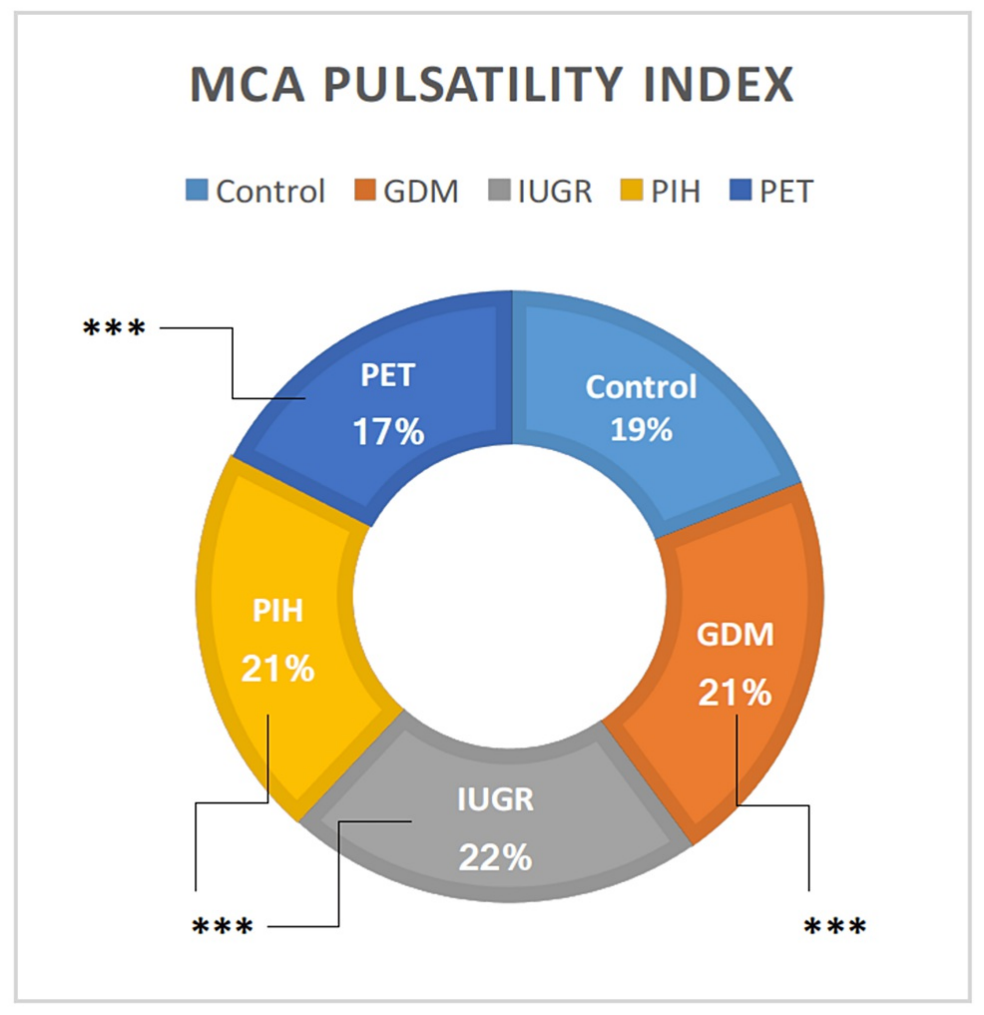
Doppler indices of MCA in high-risk groups in comparison with the control group. MCA = middle cerebral artery; GDM = gestational diabetes mellitus; PIH = pregnancy-induced hypertension; PET = preeclampsia toxemia; IUGR = intrauterine growth restriction

There was an extremely considerable distinction in the MCA PI among the groups, as seen in the ANOVA test. The high-risk groups including GDM, IUGR, and PIH showed a highly significant elevation in MCA PI than the normal pregnant women (t = 14.24, p = 0.00001; t = 12.40, p = 0.00001, and t = 10.87, p = 0.00001, respectively) but it was significantly reduced in PET group individuals than normal individuals (t = 6.670, p = 0.00001) (Table 1). The MCA PI was observed between the high-risk cohorts. There was no significant difference between the high-risk groups. GDM vs. PET, IUGR vs. PIH, IUGR vs. PET, and PIH vs. PET showed statically significant differences (t = 11.26, p = 0.00001; t = 2.48, p = 0.007489; t = 10.052, p = 0.00001; and t = 9.055, p = 0.00001, respectively), while the rest did not show a significant difference (Figure 7).

CP ratio

The one-way ANOVA results showed a significant difference in the CP ratio among the groups. The CP ratio was examined between the groups. GDM and IUGR groups showed substantially higher CP ratios than normal individuals (t = 3.35, p = 0.000448 and t = 3.27, p = 0.000609, respectively) whereas PET exhibited a lower CP ratio than the control group (t = 17.15, p = 0.00001) (Table 1). However, PIH showed no difference when compared with the control group. The GDM vs. IUGR was not significant (t = 0.36, p = 0.358216), whereas GDM vs. PIH, GDM vs. PET, IUGR vs. PIH, IUGR vs. PET, and PIH vs. PET exhibited significant differences (t = 2.01, p = 0.022949; t = 10.94, p = 0.00001; t = 2.00, p = 0.024091; t = 10.20, p = 0.00001; and t = 9.75, p = 0.00001, respectively).

Fetal birth weight

The one-way ANOVA results showed a significant difference in fetal birth weight among the groups. The fetal birth weight was examined between the groups. The IUGR and PIH groups showed substantially lower fetal birth weight than normal individuals (t = 3.02, p = 0.001367 and t = 1.84, p = 0.032781, respectively), whereas the GDM and PET groups did not demonstrate considerable differences statistically (t = 0.04, p = 0.480263 and t = 1.41, p = 0.07882) (Table 1). The PIH vs. PET was not significant (t = 0.49, p = 0.310449), whereas GDM vs. IUGR, GDM vs. PIH, GDM vs. PET, IUGR vs. PIH, and IUGR vs. PET exhibited statistically considerable variation (t = 13.28, p = 0.00001; t = 7.00, p = 0.00001; t = 6.17, p = 0.00001; t = 8.59, p = 0.00001; and t = 7.74, p = 0.00001, respectively).

Luminal area umbilical vessels

**Figure 8:**
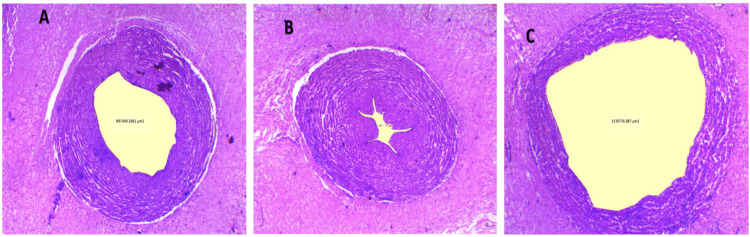
Representative photomicrographs of umbilical vessel sections of the preeclampsia group stained with hematoxylin and eosin (4× magnification). (A) Umbilical artery 1. (B) Umbilical artery 2. (C) Umbilical vein.

Using histopathological techniques, the tissue of the umbilical cord was fixed in a 10% formalin solution and then sectioned at 4 μ thickness. Staining was done using normal hematoxylin and eosin, slides were observed using a biological microscope Leica DM500, and luminal areas were measured using Leica LAS software (Figure 8).

Luminal Area Umbilical Artery 1

**Figure 9:**
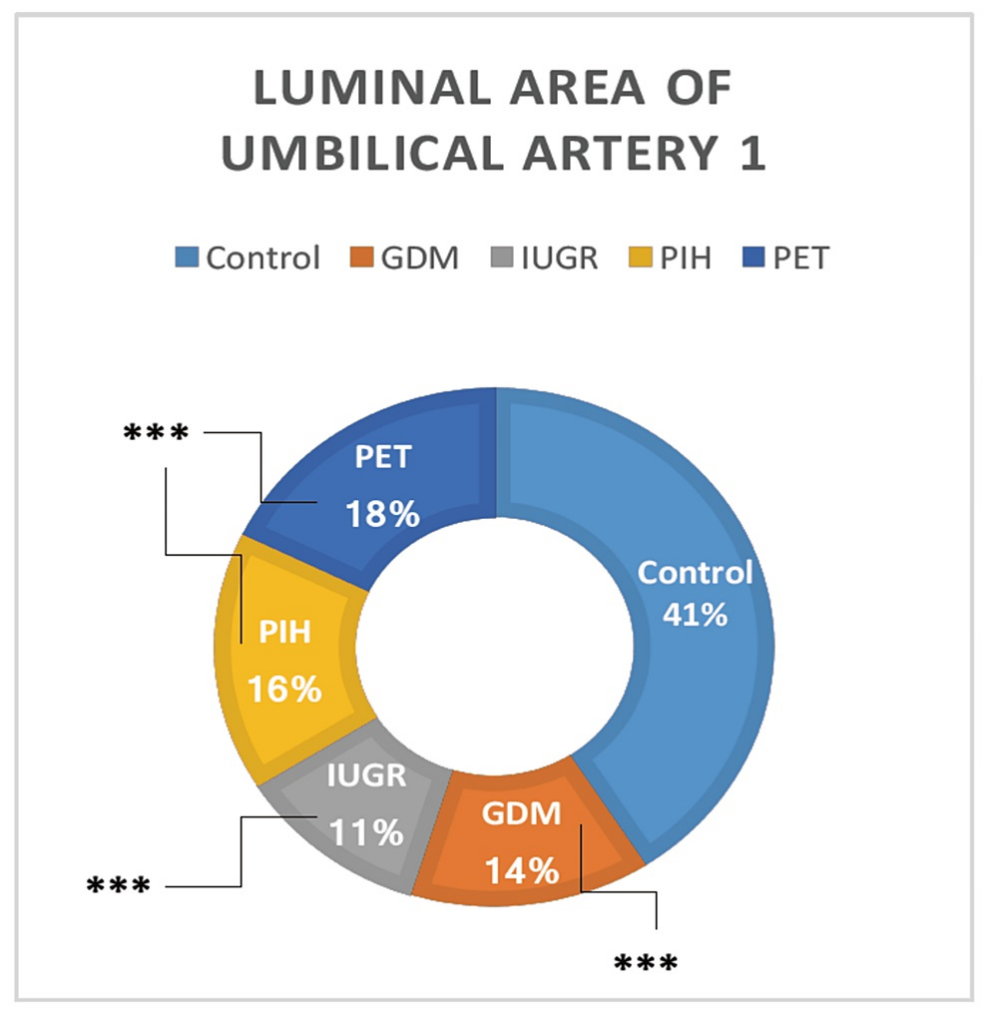
The luminal diameter of the umbilical artery 1 in different high-risk groups in comparison with the control group. GDM = gestational diabetes mellitus; PIH = pregnancy-induced hypertension; PET = preeclampsia toxemia; IUGR = intrauterine growth restriction

There was a highly significant variation in luminal area umbilical artery 1 among the groups, as tested by ANOVA. All high-risk groups (GDM, IUGR, PIH, and PET) showed a highly significant reduction in the luminal area of the umbilical artery 1 than the normal pregnant women (t = 4.50, p = 0.000013; t = 3.15, p = 0.001246; t = 4.06, p = 0.000063; and t = 3.37, p = 0.000624, respectively) (Table 1). The luminal area umbilical artery 1 was observed between the high-risk groups and no significant difference was detected (Figure 9).

Luminal Area Umbilical Artery 2

**Figure 10:**
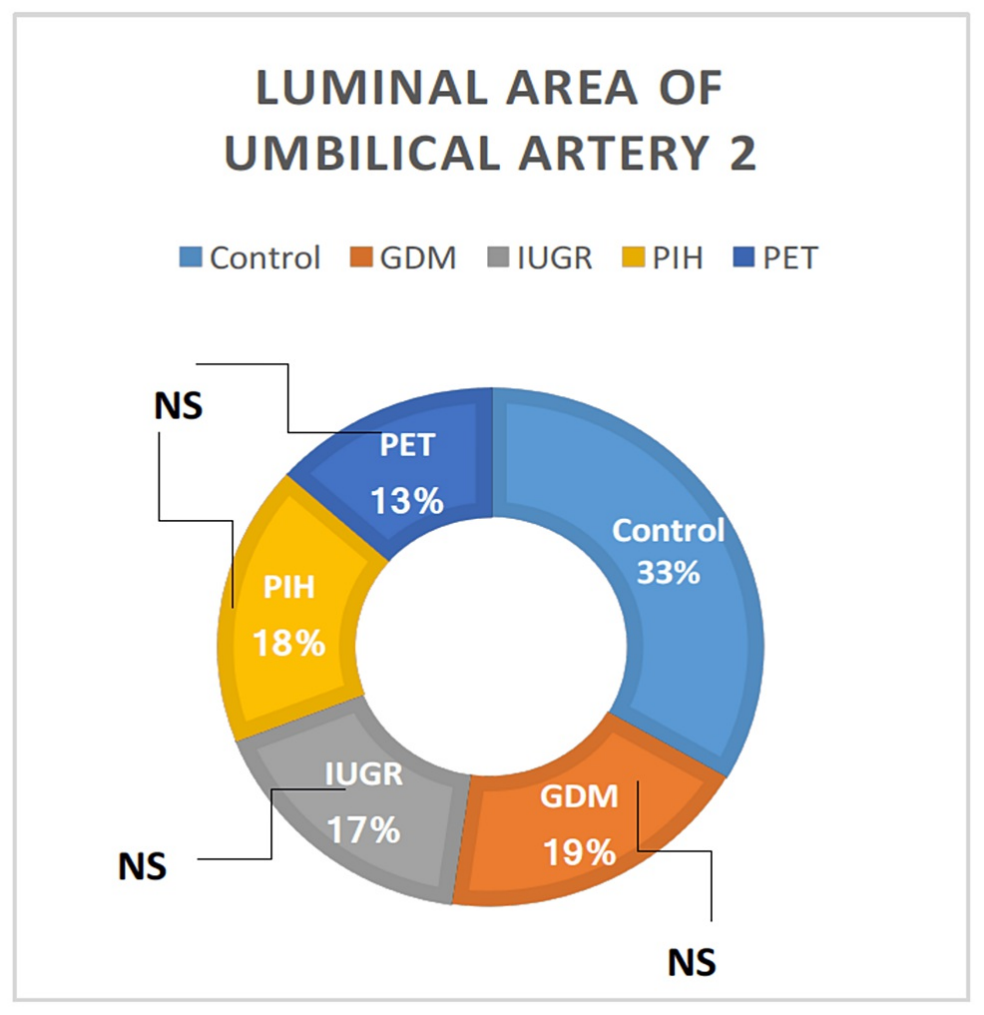
The luminal diameter of the umbilical artery 2 in different high-risk groups in comparison with the control group. GDM = gestational diabetes mellitus; PIH = pregnancy-induced hypertension; PET = preeclampsia toxemia; IUGR = intrauterine growth restriction

There was no significant variation in luminal area umbilical artery 2 among the groups, as tested by ANOVA. All high-risk groups (GDM, IUGR, PIH, and PET) showed a relative reduction in luminal area umbilical artery 2 than the normal pregnant women but it was not statistically significant because of the high standard deviation of means (Table 1). The luminal area umbilical artery 2 was observed between the high-risk groups and no significant difference was noted (Figure 10).

Luminal Area Umbilical Vein

**Figure 11:**
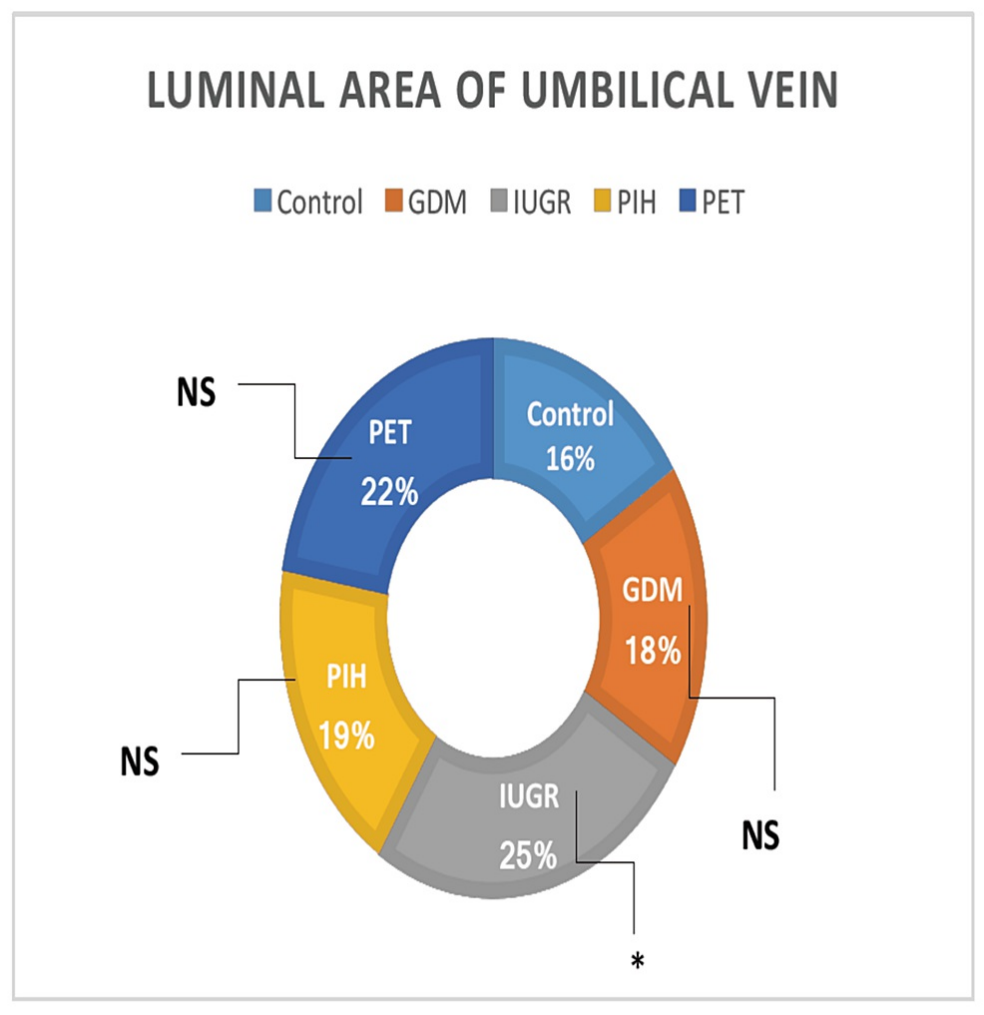
The luminal diameter of the umbilical vein in different high-risk groups in comparison with the control group. GDM = gestational diabetes mellitus; PIH = pregnancy-induced hypertension; PET = preeclampsia toxemia; IUGR = intrauterine growth restriction

There was no significant variation in the luminal area umbilical vein among the groups, as tested by ANOVA. All high-risk groups (GDM, PIH, and PET) showed relatively equal luminal area umbilical veins than the normal pregnant women, except the IUGR group which showed higher luminal area umbilical veins, but it was not statistically significant because of the high standard deviation of means (Table 1). The luminal area umbilical vein was observed between the high-risk groups and no significant difference was noted (Figure 11).

## Discussion

The placenta is an essential structure for nutrients and metabolite exchange between the mother and conceptus. Normal umbilical cord attachment occurs in the middle of the fetal aspect of the placenta. The length of the stem villi decides the thickness of the placenta. Hyperplasia and hypertrophy are the two stages of the placenta’s growth and development [17]. The results exhibited that except for the GDM group, the rest of the groups (IUGR, PIH, and PET) showed a highly significant reduction in the estimated placental weight and the actual placental weight than normal pregnant women. It is in line with an earlier study conducted by McNamara et al. (2014) [8] who advocated that reduced weight of the placenta was linked to persistent hypertension, but preeclampsia was only linked to low placenta weight before birth weight adjustment. Placental volume weight of the baby and placenta were reduced in the groups with PET, GDM, and SGA than the control cohort [2].

The present study results demonstrated that placental thickness was reduced in all high-risk groups, but the GDM and preeclampsia groups showed no distinctive differences. According to the study by Sun et al. (2021) [20], the elevated thickness of the placenta might be a strong prognosticator of high-risk pregnancies, particularly those with PET, hydrops fetalis, and GDM [18], which is in line with the current study findings where there was a rise in the number of blood vessels per placental villous in diabetes individuals. It is because of increased neoangiogenesis in diabetic individuals. All of these blood arteries had thicker walls despite being young and several had fibrinoid thrombi. Some of the villous blood arteries in the diabetic placentae were found in the center of the villi. As a result, the placental barrier’s thickness was raised in the placentae of diabetics [19]. The IUGR group exhibited a drastic reduction in placental thickness followed by PIH. Generally, the placental thickness should be 10 mm, or equivalent to the fetal age in weeks, as the thickness of the placenta rises throughout pregnancy [20]. The prevalence of both SGA and large for gestational age (LGA) fetuses, hydrops fetalis, and greater perinatal death have all been linked to the thickness of the placenta [21,22]. The present results showed no distinction in the thickness of the placenta in the GDM and PET groups in normal pregnant women. However, the PIH and PET groups showed significantly lower placental diameters. The least placental diameter was found in IUGR followed by PIH group individuals.

According to previous investigations, the number of cotyledons is much larger in the placentas of GDM women than in non-GDM mothers. In contrast to the non-GDM group, the GDM group placentas simultaneously increased in width and weight, which may have been an adaptive response [23]. Our study results support the aforesaid research findings. The placental cotyledon count except for the GDM group was higher but did not show statistical significance. The rest of the groups (IUGR, PIH, and PET) showed a reduction in the number of cotyledons than normal pregnant women, which is in line with existing literature. Preeclampsia caused an inadequate blood supply, which was reflected in the preeclampsia group’s smaller placentae's diameter, thickness, number of cotyledons, and volume [24]. Preeclamptic pregnancies had considerably lower fetal birth weights and placental weights, diameters, and cotyledon counts than normotensive pregnancies [25]. The IUGR group showed the least placental cotyledon count than the rest of the groups, followed by the PIH and PET groups. As a result of their increased risk of hypoxemia, IUGR infants with placental insufficiency because of the reduced number of cotyledons are less likely to withstand labor and are more likely to lead to birth through cesarean sections [26].

In a healthy pregnancy, placental weight, fetal weight, and the fetoplacental weight ratio all increase gradually with gestational age, with fetal weight increasing more quickly than the weight gain of the placenta. After the fetus outgrows the placenta at 42 weeks of gestational age, the fetoplacental ratio rises gradually at first before rising suddenly at 43 weeks of gestational age. Gestational age greatly influences fetal weight, placental weight, and fetoplacental ratios [27]. The fetoplacental ratio rises in the group of normal pregnant women [27], but in this study, we did not find any significant alteration in high-risk groups when compared with the control group.

In contrast to the non-GDM group, the umbilical cord length was shorter in the GDM cohort [28]. Gestational hypertension was linked to umbilical cord anomalies, such as aberrant length, diameter, insertion, entanglements, knots, and coils [29], but this study’s results did not show any significant difference in high-risk groups when compared to the control group.

The fetal circulatory alterations that result from hypoxia include elevated impedance in umbilical veins and decreased impedance in cerebral arteries. Less maternal cardiac output and greater peripheral vascular resistance are related to these modifications. When umbilical Doppler results are seriously aberrant, this becomes especially clear. To ameliorate pathologically aberrant uteroplacental function and, therefore, fetal state, this link opens the possibility of therapeutic treatment of maternal cardiovascular function [30].

The study results revealed that all high-risk groups including GDM, IUGR, PIH, and PET showed a highly significant elevation in UA PI than normal pregnant women. The high-risk groups including GDM, IUGR, and PIH showed a highly significant elevation in MCA PI than normal pregnant women, but it was significantly reduced in PET group individuals than normal individuals. According to Leung et al. [30], neither UA PI nor MCA PI helped identify an aberrant pregnancy outcome in GDM [31]. While Niromanesh et al. [31] claimed that faulty UA Doppler evaluation is associated with poor newborn outcomes [32]. Additionally, Shabani Zanjani et al. [32] emphasized that individuals with GDM had higher MCA PI values [33]. Further, earlier research did not assess standardized color Doppler ultrasound (CDUS) characteristics; for instance, certain investigations assessed only PI values, while others focused on the CP ratio, etc. Besides, the majority of the investigations that were published in the scientific literature used CDUS data that were collected during third-trimester assessments. Moreover, Niromanesh et al. (2017) emphasized the usefulness of UA and MCA CDUS alterations in prognosticating poor infant outcomes in the GDM cohort. They used a different approach and assessed how CDUS alterations affected the course of the pregnancy. Instead of defining CDUS characteristics separately, they simply characterized UA and MCA examinations as normal or abnormal. They also did not provide a certain timing for CDUS evaluation [32].

The values of mean PI, RI, and SD were substantially greater in umbilical artery IUGR patients than in non-IUGR instances, whereas the values of MCA PI, RI, and SD were considerably less in IUGR cases than in non-IUGR individuals [33]. According to earlier investigations, cases with severe PIH had a considerably higher mean UA PI than individuals with moderate PIH [34], which is similar to the current study. However, the PI values of fetal MCA were considerably lower among PIH patients [35], which is in contrast with the results of this study. Similar fluctuation in Doppler indicators with the severity of the disorders was also seen in research on preeclampsia and prenatal hypertension individuals. As gestational age increased, the normal ratios of MCA/UA PI dropped. Overall, 30% of moderate instances and 46% of severe cases of preeclampsia exhibited fetal circulation, as evidenced by a low ratio of MCA/UA PI [36].

In comparison to healthy fetuses, Singh et al. (2013) [37] found that IUGR fetuses had higher Doppler indices. According to Sattar et al. (2011), in comparison to the normal group, the IUGR-suspected fetuses group had higher values of Doppler PI [38]. Their conclusions of Doppler findings are akin to the current investigation.

Low CPR and the perinatal outcomes of pregnancies affected by a hypertension condition are related. Compared to other forms of hypertension diseases, this association seemed to be greater in PET [39], which is in line with our observation. In comparison to the control groups, the CP ratio in the GDM and IUGR groups revealed markedly greater values, whereas PET showed lower values. However, due to fetal discomfort and a composite unfavorable perinatal outcome, lower CPR is linked to a greater probability of obstetric intervention [40], this change we observed only in the PET group.

While the GDM and PET groups did not show statistical significance in the birth weight of newborns compared with control. However, the IUGR and PIH groups showed a substantial reduction in fetal birth weight. The GDM group demonstrated a greater fetal weight, which is consistent with past results. The risk of LGA and greater birth weight were both significantly enhanced by GDM. Post-load glucose levels had more of an impact on fetal development than fasting blood glucose. Additionally, the birth weight, likelihood of LGA, and macrosomia were significantly affected by the blood glucose levels at various time points [41]. A decrease in birth weight was correlated with preeclampsia [42]. Low birth weight and IUGR are both made more likely by preeclampsia [43].

Preterm births were more common in preeclamptic women (26.7%). Preeclamptic mothers gave birth to babies with lower birth weights, lengths, and head circumferences. Significant statistical contributions to SGA were made by severe preeclampsia. The hypoperfusion model was utilized to explain the pathophysiology of preeclampsia in PIH mothers who had lower birth weight babies. When uteroplacental perfusion decreased owing to preeclamptic women’s condition, LGA children were delivered as a result of compensating illnesses such as GDM or obesity in mothers [44].

Infants with IUGR or SGA status at delivery are more likely to die during pregnancy and have birth-related complications, such as acidosis during the perinatal period, hypothermia, coagulation abnormalities, hypoglycemia, and specific immunologic issues. Along with chronic lung disorders as well as necrotizing enterocolitis, babies with IUGR appear to be more vulnerable to other prematurity-related problems. The effects of IUGR on children include a small but considerable rise in the risk of neurological problems such as cerebral palsy as well as an increased risk of short stature, cognitive delays, and compromised performance in school [45]. A newborn with a low birth weight is born at full term weighing less than 2,500 g [46]. It may be the result of premature delivery or IUGR is frequently a comorbidity of preterm birth and is correlated with both the aided and unassisted induction of preterm delivery [47,48].

All high-risk groups (GDM, IUGR, PIH, and PET) showed a highly significant reduction in the luminal area umbilical artery 1 than normal pregnant women. Luminal area umbilical artery 2 was also lower in high-risk groups than control, but it was not statistically significant because of the high standard deviation of means. All high-risk groups showed relatively equal luminal area umbilical veins than the normal pregnant women, except the IUGR group which showed a higher luminal area umbilical vein, but it was not statistically significant because of the high standard deviation of means. We could not find any earlier research data related to the luminal area of umbilical vessels.

The study aims to offer a comprehensive understanding of the relationships between Doppler indices and placental/neonatal morphometry in various pregnancy complications and normal pregnancies. The findings from this study could have implications for better understanding the pathophysiology of pregnancy complications and for potentially improving clinical management strategies. Limitations, such as the cross-sectional design and potential confounding variables, should be acknowledged. Future studies will consider subgroup analyses based on the severity and timing of pregnancy complications if feasible.

## Conclusions

This study enriches the understanding of the interrelationships among Doppler indices, placental parameters, and high-risk pregnancies, offering a comparative lens by including normotensive pregnancies. Current evidence suggests that the use of Doppler ultrasound on the UA in high-risk pregnancies reduces the risk of perinatal deaths and may result in fewer obstetric interventions. The results should be interpreted with caution. By shedding light on the intricate dynamics at play, the study paves the way for improved maternal and fetal care, providing a foundation for evidence-based clinical decisions within the realm of high-risk obstetrics.
